# High perceived stress in patients on opioid agonist therapies during rapid transitional response to the COVID-19 pandemic in Ukraine

**DOI:** 10.3389/fpubh.2023.1231581

**Published:** 2023-11-30

**Authors:** Samy J. Galvez, Frederick L. Altice, Anna Meteliuk, Roman Ivasiy, Eteri Machavariani, Scott O. Farnum, Tetiana Fomenko, Zahedul Islam, Lynn M. Madden

**Affiliations:** ^1^Yale School of Medicine, Section of Infectious Diseases, New Haven, CT, United States; ^2^Division of Epidemiology of Microbial Diseases, Yale School of Public Health, New Haven, CT, United States; ^3^APT Foundation, New Haven, CT, United States; ^4^Alliance for Public Health of Ukraine, Kyiv, Ukraine

**Keywords:** opioid agonist therapies, methadone, buprenorphine, stress, COVID-19, Ukraine

## Abstract

**Background:**

The COVID-19 pandemic resulted in marked disruptions in healthcare delivery in Ukraine related to emergency guidance in response to treating opioid use disorder (OUD). Patients with OUD, a group with high levels of comorbid medical and psychiatric disorders, and prescribed opioid agonist therapies (OAT) were rapidly shifted to take-home dosing if they were deemed clinically stable. The impact of these shifts on patient stress and related substance use during the pandemic, however, is unknown.

**Methods:**

In early May 2020, 269 randomly selected OAT patients in Ukraine were surveyed to assess their stress level and substance use using the validated Perceived Stress Scale and examined correlates of severe perceived stress.

**Results:**

Overall, 195 (72.5%) met criteria for moderate to severe levels of stress, which was independently correlated with having started OAT within the past 12 months (aOR: 1.33; 95%CI: 1.15–1.55), living in a large metropolitan area (aOR: 1.31; 95%CI: 1.18–1.46), having been asked by others to share their medication (aOR: 1.13; 95%CI: 1.02–1.25), and having an increase of over 10 min in transportation time to get to treatment (aOR: 1.16; 95%CI: 1.04–1.29). Twenty seven (10%) patients felt at high risk of relapse, while 24 (8.9%) patients reported purchasing drugs.

**Conclusion:**

During a time of great uncertainty soon after emergency guidance to the COVID-19 pandemic, there was extraordinary high levels of perceived stress reported. In response to emergency guidance, OAT patients should be screened for perceived stress and certain subgroups should be targeted for additional psychosocial support.

## Introduction

The unprecedented impact to health systems due to the COVID-19 pandemic has introduced new challenges and increased the risk for people with opioid use disorder (OUD), who are particularly vulnerable to stress—a documented psychological effect of the pandemic (1–3). Stress has emerged as one of the main psychological consequences to the COVID-19 pandemic, as individuals become fearful of COVID-19 infection, socioeconomic consequences, and traumatic stress symptoms, among other stress-induced negative effects (4, 5). In some populations, over half of all individuals have reported the adverse psychological consequences of COVID-19 as moderate to severe (6). For people with OUD, stress has long been associated with more negative treatment outcomes, potentially influencing opioid agonist therapy (OAT) at every step along the opioid treatment cascade by contributing to treatment dropout (7), craving, and substance use relapse or increased use among people with OUD (7–9).

Ukraine is a middle-income country in the Eastern Europe and Central Asian (EECA) region (10), the largest region globally where both new HIV infections and HIV-related mortality continues to rise, fueled primarily by people who inject drugs (PWID)—mostly of opioids (11, 12). Ukraine introduced OAT in 2004, primarily as HIV prevention (13), yet scale-up has been slow, constrained by patient, clinic, and policy factors (14–20). The first case of COVID-19 in Ukraine was diagnosed on March 3, 2020, followed by the first death 15 days later. At the onset of the pandemic, there were 12,837 people receiving OAT in Ukraine, representing only 2.9% of all PWID in Ukraine, an improvement from previous years but still falling well below the minimum 20% coverage level recommended to most efficiently reduce HIV transmission in the country (21). In response to the pandemic, narcologists (addiction treatment specialists) followed guidance from the country’s Center for Public Health to identify and transfer a significant number of their stable patients from daily, in-clinic, methadone dosing to 7- and 10-day take-home doses (22).

While reducing program demands on patients in treatment may have reduced COVID-19 transmission by reducing daily in-person interactions between clinicians and patients, narcologists remained concerned about the possible psychological effects of reduced interpersonal contact and oversight with their OAT patients. Psychiatric disorders, especially mood disorders, are particularly prevalent in Ukraine (23). This circumstance is exacerbated by ongoing conflict in the Crimea and Donbas regions (24), political unrest, and economic insecurity. Such disorders are distinctly more prevalent in OAT patients (50–64%) relative to the general population (25). At the same time, these patients experience less access than non-OUD patients to psychiatric treatment (including therapy and medication). This is occurring in a country where access to mental health and psychiatric treatment and services is limited (26) and stigmatized (27). Furthermore, the economic impact of COVID-19 increased isolation due to physical distancing measures, uncertainty about an ongoing pandemic, fear of contracting COVID-19, traumatic stress symptoms as a result of direct or indirect exposure to COVID-19, and a constantly changing social environment, each of which may magnify stress. These factors potentially impact the psychological well-being of patients on OAT (4, 8, 28, 29). In our exploration of the stress status of OAT patients in Ukraine, we seek to understand the effects of the COVID-19 pandemic on the perceived stress among OAT patients by examining their exposure to stress-inducing circumstances and related substance use in response to an evolving pandemic during the initial phases.

## Methods

For this study, we randomly surveyed 269 participants by telephone from May 4–15, 2020 by trained interviewers from the 12 administrative regions of Ukraine that have more than 400 patients on OAT (Kyiv, Vinnytsia, Dnipro, Kryvyi Rig, Donetsk, Zaporizhzhia, Kirovograd, Poltava, Odesa, Sumy, Mykolayiv and Cherkasy) using the Perceived Stress Scale (PSS) along with tailored questions regarding stressors and behaviors related to having OUD and drug use. Twenty OAT patients were randomly selected from each of the participating regions using the SYREX database – a national register of all OAT patients in Ukraine. Subsequently, individuals participating in methadone therapy were selected through a random sampling procedure. Using the EpiTools Sample Size Calculator, a prevalence of 0.2 and precision of 0.05 in the known number of patients on OAT suggested that 246 participants were needed (30). Upon their arrival for methadone treatment, an attending nurse extended an invitation to these patients to partake in the research study. In the event of an affirmative response, patients were directed to a research assistant, where they anonymously underwent the informed consent process and completed the survey. Notably, there were no instances of patient refusal among those solicited for survey participation. Each phone survey took about 15 min. The interview included 3 sections: (1) socio-demographic information (age, gender, education, employment and marital status); (2) the PSS (31), which was translated and back-translated for cultural understanding (32); and (3) behavioral responses during the pandemic. After initially sampling 255 participants, we purposefully sampled an additional 14 patients at random from these sites to ensure enough new patients to OAT (≤12 months), given the known association between stress and recent treatment initiation and to ensure that our sample was representative of OAT patients in Ukraine (8). Data were analyzed utilizing the R statistical software, with the addition of the “plyr,” “MASS,” and “psy” packages. After initial scoring of the PSS instrument, variables were recoded for analysis as factors in a logistic regression. The primary outcome was moderate to severe perceived stress, defined as having a score > 13. Independent variables were primarily dichotomized. For the change in time to get to the OAT clinic, the variable was dichotomized at the median (10 min more travel time). Multivariable logarithmic regression analysis was conducted to establish the independent correlates of reporting moderate to severe stress levels. Only statistically significant variables (*p* ≤ 0.05) were included in the final model. Inclusion of non-significant variables in the final model did not change the final outcomes.

### Ethical considerations

At the beginning of the phone interview, each participant was provided with detailed description of research and provided oral informed consent before the survey began. All study tools and ethical oversight were approved by the Institutional Review Board at the Alliance for Public Health in Kyiv, Ukraine.

## Results

Participant characteristics (*N* = 269) are reported in Table 1. Participants were primarily from highly populated areas, with two thirds of participants living in cities with over one million inhabitants. The mean age was 40 years (sd = 8.8) with an average of 72 (sd = 52.9) months on OAT, with 14% of all participants having initiated OAT within the last 12 months. Approximately half of those interviewed were married and over 56% of them were unemployed. These findings are similar to other studies of PWID on OAT (33). The mean PSS score was 16.9 (sd = 6.3), with 27.5% (*n* = 74) meeting criteria for low-perceived stress, 68.4% (*n* = 184) for moderate perceived stress, and 4.1% (*n* = 11) for severe perceived stress.

**Table 1:** Participant characteristics (*N* = 269).

Variables	N	%
Region size		
>1 million	178	66.2
< 1 million	91	33.8
Mean age, years	40	10*
Sex		
Male	191	71.0
Female	78	29.0
Education		
Secondary or less	83	30.9
Beyond secondary	186	69.1
Marriage status		
Married	126	46.8
Not married	143	53.2
Employment status		
Employed	118	43.9
Unemployed	151	56.1
Time on OAT		
<12 months	38	14.1
**≥**12 months	231	85.9
Increase in time to clinic of over 10 min		
yes	109	40.5
no	160	59.5
Perceived stress category		
low stress	74	27.5
moderate to severe stress	195	72.5
Has been asked to share drugs since the beginning of the COVID-19 pandemic		
Yes	86	32.0
No	183	68.0
Perceived risk of relapse relative to before COVID-19		
Higher	27	10.0
Similar or lower	242	90.0
Bought drugs since COVID-1		
Bought drugs	24	8.9
Did not buy drugs	245	91.1

OAT, opioid agonist therapy. *Indicates the Inter-Quartile Range.

Participants reported a median increase of 10 (IQR: 0–25) minutes in travel time to their OAT dispensing site due to transportation restrictions imposed by the Ukrainian government during the COVID-19 pandemic, with 40% of them reporting an increase in travel time of over 10 min. Additionally, approximately one third (*n* = 86) reported having been asked to share their OAT medication during the COVID-19 quarantine, and 8.9% (*n* = 24) reported buying drugs during the quarantine, with the large majority of those who reported having bought drugs stating that they were not any more difficult to obtain than the time before the quarantine (*n* = 17). Despite the changes that emerged during the early COVID-19 restrictions, only 10.0% (*n* = 27) of all participants reported feeling at a higher risk of relapse due to the pandemic and its ensuing challenges—the large majority felt their risk of relapse remained the same and 24 (8.9%) had purchased drugs while on OAT.

Multiple regression analysis findings are reported in Table 2. Overall, 195 (72.5%) met criteria for moderate to severe perceived stress, which was independently correlated having started OAT within the past 12 months (aOR: 1.33; 95%CI: 1.15–1.55), living in a large metropolitan area (aOR: 1.31; 95%CI: 1.18–1.46), having been asked to share their treatment medication (aOR: 1.16; 95%CI: 1.04–1.29), and having an increase of over 10 min in travel time to get to the OAT clinic for treatment (aOR: 1.13; 95%CI: 1.02–1.25). Being unemployed resulted as a variable in the final, fitted model, but its effect on the outcome was not significant.

**Table 2:** Independent correlates of having severe perceived stress levels (*N* = 269).

Variables	uOR	95% CI	*p* value	aOR	95% CI	*p* value
Intercept	1.24	0.86, 1.79	0.248	1.42	1.25, 1.62	<0.001
Large Metro Area (> 1 million)	1.31	1.17, 1.46	<0.001	1.31	1.18, 1.46	<0.001
Age	1.00	0.99, 1.01	0.556	–	–	–
Sex (Male)	1.05	0.94, 1.18	0.368	–	–	–
New to OAT	1.35	1.12, 1.62	0.002	1.33	1.15, 1.55	<0.001
Did not complete secondary education	0.99	0.87, 1.11	0.876	–	–	–
Not Being Married	1.07	0.96, 1.19	0.206	–	–	–
Being Unemployed	1.11	0.99, 1.23	0.074	1.09	0.98, 1.22	0.100
Increased time to OAT Clinic (10+ min)	1.13	1.02, 1.26	0.021	1.13	1.02, 1.25	0.020
Asked to Share Drugs	1.15	1.02, 1.29	0.023	1.16	1.04, 1.29	0.011
High Risk for Relapse Perception	1.04	0.84, 1.27	0.737	–	–	–
Has Bought Drugs	1.00	0.82, 1.23	0.977	–	–	–

uOR, unadjusted Odds Ratio; CI, Confidence Interval; aOR, adjusted Odds Ration; OAT, Opioid Agonist Therapy.

## Discussion

Similar to findings of the initial COVID-19 outbreak in China, perceived stress (6) and generalized anxiety disorders (5) were high. The levels of perceived moderate to severe stress among patients with OUD, however, were markedly higher among oud study cohort than reported in the general population. This is especially concerning since a report from the Well Being Trust suggested that economic and employment consequences of COVID-19 could result in markedly higher levels of drug and alcohol use, resulting in markedly elevated levels of “deaths of despair” (34). These negative outcomes are even more likely in a population prone to drug misuse reported elsewhere (35, 36). In this study, the concomitant concern about and actual purchase of drugs was relatively low and similar to reports from OAT patients in Ukraine long before the pandemic (37). In people with OUD, and especially those in treatment in which they must travel regularly (for many, daily travel is required for supervised dosing) to a medical clinic in person to receive their medications, the risk to these individuals for acquiring COVID-19 is heightened. One report from the United States suggests that people with OUD are at over a 10-fold elevated risk for becoming infected with COVID-19 (38), which may contribute markedly to stress levels in OAT patients.

There are several important findings from this study that merit attention, not only for Ukraine, a country with an extraordinary level of OUD, but for other countries that are experiencing a more profound COVID-19 epidemic. In fact, the COVID-19 epidemic was not nearly as severe in Ukraine as in Western Europe and North America at the time of the survey. Findings from the multivariable analysis provide some important insights into who to target for screening for stress and then prioritize them for supplemental psychosocial support. This is especially important given the extraordinarily high proportion of OAT patients reporting moderate to severe perceived stress. The rationale for intervening with OAT patients, especially those who are relatively new to OAT, are vulnerable to relapse to drug or alcohol use and consequently at elevated risk for overdose. In this study, though there is no matched comparison to drug use in the pre-COVID period, the level of self-reported drug use was not high. Explanations for the low level of drug use may include social desirability bias or that patients were less vulnerable because they were prescribed optimal dosages of methadone. Though the methadone dose was not reported in this survey, a large proportion of patients on OAT in Ukraine receive optimal dosing (33), which may have been sufficient to reduce cravings even in the presence of high perceived stress. Moreover, changes in life circumstances can contribute to stress. For example, most patients in our sample had increased travel time to receive their medications (sometimes daily). Increased travel time of 10 min or more correlated with higher severe stress levels. While other studies have shown that stress and anxiety levels are generally higher in more urban settings (perhaps related to where most initial COVID-19 cases occurred), it was generally found to be the case elsewhere during COVID-19 where crowding and increased potential for exposure was of concern (5).

There are potential explanations for higher perceived stress among those who were asked to share their OUD medications; first, it may be the case that those with severe stress were still spending time in the “drug scene” where peers felt comfortable asking them to share their methadone. Alternatively, patients who were relatively stable yet asked to share their medications may have perceived more stress because their sobriety may have been put at increased risk if they shared. Certainly the situation of being asked to share a prescribed medication with others is a nuanced conflict for a person who is trying to abstain from opioid use.

Despite the high levels of stress reported by OAT patients, findings here point to a number of observations that bode well for the country and those using MOUD. First, despite the high perceived stress, few had relapsed or continue to use drugs. Whether this finding persisted longer into the epidemic requires further analysis. Initial data, however, shows that relapse rates have remained similar to pre-pandemic rates (22) signaling how, despite high perceived stress, patients can continue to manage their medications adequately. Patients may have been more successful in self-regulating their stress and relapse potential because they were free from other stressors such as travel to treatment, which is often associated with police harassment (39, 40), or felt like they were at reduced risk for COVID-19 by reducing their community exposure. Second, despite high stress levels, OAT scale-up continues toward its national goals throughout COVID-19 restrictions with Ukraine achieving its highest gains in OAT scale-up in any given year (41). Successful OAT scale-up with decreased in-person interactions with take-home dosing and no increase in mortality suggest that both clinical or self-management remains adequate (22).

Though we did not measure to what extent patients in this survey received tele-health to support them during this observation period, the sheer magnitude of severe perceived stress levels by OAT patients provides information about strategies that might benefit patients when in-person contact decrease. The findings herein suggest that strategies targeted to relatively new patients in more urban settings and those who must increase their transit to OAT clinics might benefit from routine screening for stress combined with evaluation and treatment counseling using tele-health. For example, scripted telephone calls with brief counseling oriented toward managing daily challenges may reduce stress in OAT patients. A review of self-guided and clinician-guided tele-counseling strategies have demonstrated effectiveness at reducing stress during COVID-19 (42), but such strategies with OAT patients is understudied (1). Other mHealth strategies (including tele-health) may prove useful with stress management in patients with OUD (43). Adaptations for OAT clinicians might include tips on how to manage medication at home (i.e., keeping it out of the hands of others), how to respond to requests to share medication (and the consequences of personal withdrawal), how to remain socially, if not physically engaged, how to recognize and prevent overdose, how to guide peers facing similar challenges, and other targeted topics. Although not studied among individuals with OUD, these types of phone calls have been previously implemented successfully to aid in self-management of stress due to chronic conditions (44–46). The frequency of such calls may be even more helpful in urban settings, where patients experience higher stress levels.

Priority calls and or contact should be given to those newly initiated into OAT, as they may not have experience managing medications, may not yet be accustomed to side effects, and are currently experiencing higher levels of stress. Providing additional flexibility regarding hours of operation and availability of appointments for patients new to OAT may aid in allowing them more options in treatment. Given previously found associations between social support and reduced likelihood of drop-out (9, 28, 47), interventions involving close family members or friends may be considered to aid new patients. Additional flexibility regarding dosing for patients newly initiated into OAT should also be considered as they may require more frequent assessment of their dose. Engaging social workers to aid in improving the employment status of patients may also be a promising practice, as having any employment at all (even between 1 and 10 h per week) has previously been significantly associated with lower stress levels—highlighting the clinical impact of the socioeconomic challenges introduced by the COVID-19 pandemic.

Despite the many important findings herein, there are limitations. First, we lack comparable stress perception data from the period before the pandemic. While overall surveys have shown an increase in stress levels globally during the pandemic, no specific data for this population exists to make an accurate comparison. Second, it is still too early to understand the stress trajectory of OAT patients during the pandemic, especially as the pandemic evolves with new waves. It is worth noting that this study, while highlighting substantially higher stress levels among individuals with opioid use disorder (OUD) compared to the general population, does not allow us to ascertain whether these heightened stress levels are solely attributed to OUD or if the COVID-19 pandemic itself played a significant role. We plan, however, to longitudinally assess perceived stress among OAT patients as the pandemic evolves. Understanding the primary drivers of stress among OUD patients during the pandemic would be valuable for tailoring effective interventions and support strategies. Third, we lack substantive data on access to psychiatric and mental health treatment services among this population. Of note, however, urban settings have better access to mental health services, but even then, few psychiatric services are available except for patients experiencing the most severe psychiatric symptoms or illness. Future studies should seek to understand how these services may impact and affect stress among OAT patients. Last, we limited our survey sample to sites with over 400 patients. Smaller treatment sites may have more limited services, opening hours, or numbers of providers, which could have differing effects on the treatment of their patients.

Despite the challenges faced by OAT patients and stated limitations, there are several lessons learned which may aid in ensuring sustainability of OAT in Ukraine and similar settings. Primarily, reliance on in-person visits as a requirement for treatment should be reconsidered, especially for patients who have been on treatment for over a year. These findings are counterfactual to the current legal framework for OAT that existed before COVID-19, where OAT patients had substantial personal demands on them with required daily supervision over 6 months before allowing patients to self-manage themselves with take-home dosing. As a consequence of these findings, Ukraine modified Order 200, the governmental regulation for OAT, to allow patients take-home dosing as soon as 3 months after induction with three negative monthly urine drug testing for opioids. Findings elsewhere suggest that demands on patients can be reduced without compromising safety (48). These findings further support the stability of patients, even during high-stress periods strengthening the argument to allow more self-management options for patients (49). Decreasing program-level demand for OAT delivery sites will not only increase the capacity of these sites to treat more patients, but also improve treatment outcomes for existing patients (50). As the COVID-19 crisis continues to evolve, decreasing the burden of in-person visits for patients can ease the psychological and physical burdens of existing OAT patients while allowing narcologists to devote more resources to expanding access for new patients and helping newly initiated patients during their initial period of treatment. Lastly, it remains relevant to study the effectiveness of psychosocial and behavioral interventions for stress management among patients on OAT. Current restrictions due to the COVID-19 pandemic might prevent more traditional interventions, so mHealth-based interventions remain a promising and untapped resource that could help in stress management without compromising social distancing measures or imposing a significant workload on care delivery sites. Overall, the stress management among OAT patients should be prioritized and further studied, as stress remains a significant predictor of overdose and drop-out for persons with substance use disorders.

## Conclusion

In conjunction with the COVID-19 pandemic and changes to governmental recommendations to shift patients to take-home dosing, OAT patients experience substantial levels of perceived stress in Ukraine. As the pandemic recommendations subside, important lessons learned here provide insights into future strategies to consider with OAT patients as uncertainty evolves. As OAT providers in Ukraine shifted to tele-health during the pandemic, they were remarkably resilient when shifting their services in response to Russia’s invasion of Ukraine in February 2022 (51–55). Moreover, the lessons learned from COVID-19 suggest that transformation in service delivery appears to have several advantages without marked adverse consequences. At a minimum, this study suggests the utility of reducing in-person demands on patients while also shifting to tele-health strategies. Reducing program level demands on patients will be important to scale-up efforts, but findings here suggest the need for innovations in tele-health to help ensure care remains accessible to patients, as it can increase their sense of autonomy while decreasing their stress levels and thus aiding in their psychological well-being. Implementation of brief behavioral interventions with family members and other individuals close to patients may be promising practices to reduce stress and therefore the likelihood of relapse and/or treatment dropout. Regular and targeted check-in calls may also be utilized to help patients in their sense of risk, and mHealth-based interventions, which remain unexplored. Yet, the most challenging and stress-inducing factors during the pandemic—i.e., employment, housing, and access to transportation—only highlight the importance of a multi-sectorial and multi-level approach to recovery, as well as how the disastrous nature of the pandemic and its effects on OAT patients and at-risk populations may only be starting to be seen. Furthermore, the study’s focus on patient stress and its impact on opioid addiction highlights the critical need for integrated mental health services within addiction treatment programs globally. It reinforces the significance of addressing the psychological well-being of individuals with substance use disorders and the role of stress in treatment outcomes. By prioritizing psychosocial support and behavioral interventions, healthcare systems worldwide can enhance their response to opioid addiction, reduce relapse rates, and improve the overall effectiveness of addiction treatment. These findings serve as a reminder that, even beyond the COVID-19 pandemic, comprehensive, patient-centered care remains essential for those affected by opioid use disorder worldwide.

## Data availability statement

The raw data supporting the conclusions of this article will be made available by the authors, without undue reservation.

## Ethics statement

The studies involving humans were approved by Yale University Institutional Review Board. The studies were conducted in accordance with the local legislation and institutional requirements. The participants provided their written informed consent to participate in this study.

## Author contributions

All authors listed have made a substantial, direct, and intellectual contribution to the work and approved it for publication.
