# Corrigendum: High perceived stress in patients on opioid agonist therapies during rapid transitional response to the COVID-19 pandemic in Ukraine

**DOI:** 10.3389/fpubh.2023.1359708

**Published:** 2024-01-11

**Authors:** Samy J. Galvez, Frederick L. Altice, Anna Meteliuk, Roman Ivasiy, Eteri Machavariani, Scott O. Farnum, Tetiana Fomenko, Zahedul Islam, Lynn M. Madden

**Affiliations:** ^1^Yale School of Medicine, Section of Infectious Diseases, New Haven, CT, United States; ^2^Division of Epidemiology of Microbial Diseases, Yale School of Public Health, New Haven, CT, United States; ^3^APT Foundation, New Haven, CT, United States; ^4^Alliance for Public Health of Ukraine, Kyiv, Ukraine

**Keywords:** opioid agonist therapies, methadone, buprenorphine, stress, COVID-19, Ukraine

In the published article, there was an error in the affiliations.

Instead of “^3^Alliance for Public Health of Ukraine, Kyiv, Ukraine

^4^APT Foundation, New Haven, CT, United States”,

it should be “^3^APT Foundation, New Haven, CT, United States

^4^Alliance for Public Health of Ukraine, Kyiv, Ukraine”.

In the published article, there was an error regarding the affiliations for Lynn M. Madden.

As well as having affiliation ^3^APT Foundation, New Haven, CT, United States, they should also have ^1^Yale School of Medicine, Section of Infectious Diseases, New Haven, CT, United States.

The authors apologize for this error and state that this does not change the scientific conclusions of the article in any way. The original article has been updated.

